# Costs of hospital care for hypertension in an insured population without an outpatient medicines benefit: an observational study in the Philippines

**DOI:** 10.1186/1472-6963-8-161

**Published:** 2008-07-29

**Authors:** Anita K Wagner, Madeleine Valera, Amy J Graves, Sheila Laviña, Dennis Ross-Degnan

**Affiliations:** 1 Department of Ambulatory Care and Prevention, Harvard Medical School and Harvard Pilgrim Health Care, Boston, MA, USA; 2 Philippine Health Insurance Corporation, Manila, Philippines; 3 University of the Philippines, Manila, Philippines

## Abstract

**Background:**

Hypertension is the number one attributable risk factor for death throughout the world and a major contributor to morbidity, mortality, and increasing health care expenditures in the Philippines. Lack of access to outpatient antihypertensive medicines leads to avoidable disease progression and costly inpatient admissions. We estimated the cost to the Philippine Health Insurance Corporation (PhilHealth), which generally does not cover outpatient medicines, for inpatient care for hypertension and its sequelae.

**Methods:**

Using PhilHealth inpatient claims for discharges between July 1, 2002 and December 31, 2005, we describe costs to PhilHealth for hospitalizations classified by primary discharge diagnoses into hospitalizations for hypertension; hypertensive heart and/or renal disease; other definite; and other possible consequences of untreated hypertension and assess disease trajectory for patients with more than one admission.

**Results:**

PhilHealth reimbursed US $56 million for 444,628 hospitalizations for hypertension-related diagnoses incurred by 360,016 patients during 3.5 years; 42% of admissions were for essential or secondary hypertension; 19% for hypertensive heart or renal disease; and 39% for other consequences of untreated hypertension. Among 60,659 patients admitted during the first 18 months of the study with a diagnosis of essential or secondary hypertension, 9% were hospitalized again for treatment of sequelae; older individuals (vs. =< 40 years old), men, dependents (vs. members), and those who were employed (vs. in the private membership category) were more likely to be hospitalized again; as were those whose first admission during the study period was for consequences of hypertension (vs. essential or secondary hypertension).

**Conclusion:**

Inpatient care for hypertension and its sequelae is expensive. Since many hospitalizations may be avoided with antihypertensive pharmacologic therapy, an outpatient medicines benefit may be one cost-effective policy option for PhilHealth.

## Background

Suboptimal blood pressure control is the number one attributable risk factor for death throughout the world [1]. More than a quarter of the world's adult population, nearly 1 billion, had hypertension in the year 2000, and this prevalence has been estimated to increase to 29%, or 1.56 billion, by 2025 [2]. Approximately 7.1 million deaths per year may be attributable to hypertension [1].

Hypertension has become a major contributor to morbidity, mortality, and increased health care expenditures in the Philippines. Two out of 10 Filipinos above 20 years of age, an estimated 7.76 million in 2003, have diagnosed hypertension [3]. Sixty-one deaths per 100,000 Filipinos were attributed to hypertension in 1996 [4]. If not properly treated, hypertension leads to strokes, heart attacks, heart failure, and/or kidney disease [5]. Hypertension is amenable to lifestyle changes for some patients but often requires life-long treatment with one or more antihypertensive medications. Medication treatment of hypertension can reduce the incidence of stroke (by about 35%–40%), heart attack (by 20%–25%), and heart failure (by more than 50%) [6] and thus prevent costly inpatient care for complications due to disease progression.

Given the low socio-economic status of most Filipinos – 48% of the population lives on less than US $2 per day [7] – and the high cost of medications in the country [8], regular use of antihypertensive medicines is not affordable for many patients. However, similar to other developing and transitional countries, the Philippines has made a commitment to health insurance coverage for the country's population [9,10]. The Philippine Health Insurance Corporation (PhilHealth) insures about half of the population, who are eligible through four membership categories with different contribution structures: formally employed workers, indigents, retirees, and individually paying members [10]. PhilHealth currently reimburses for inpatient care of its members (and their dependents), on a capitated basis with caps set for each type of service (room and board, medicines, laboratory services, physician fees, and operating room fees) by hospital category (primary, secondary, tertiary) and case severity (depending on hospital type, up to four levels of severity) [11]. For example, in 2006, the maximum reimbursement for a 3-day hospital stay in a primary care hospital for a case with low severity but use of daily general and specialist care was US $75; the maximum reimbursement for the most complicated case treated for three days of generalist and specialist non-surgical care at a tertiary care hospital was US $1443 [11]. The number of reimbursable hospital days is limited to 45 days per member per year and another 45 days per year to be shared by the member's dependents. PhilHealth provides very limited outpatient benefits (for maternal care and tuberculosis treatment) which do not include medication coverage for chronic conditions.

Lack of access to outpatient medicines may lead to avoidable disease progression and costly inpatient admissions to treat hypertension and its sequelae. The present study aims to estimate current costs to PhilHealth for inpatient care of hypertension. Specifically, we assessed how much PhilHealth paid in total, per hospitalization, and per patient per year for inpatient care of members hospitalized with hypertension or its sequelae. We also characterized high cost members who incurred multiple admissions for hypertension or its sequelae during the 3.5 year follow-up period and assessed the progression of their hypertensive disease over time.

## Methods

We extracted from PhilHealth inpatient claims files all hospital claims reimbursed by PhilHealth with discharge dates between July 1, 2002 and December 31, 2005 and a primary discharge diagnoses of essential or secondary hypertension or conditions that could result from untreated hypertension (Table 1). We classified possible sequelae of hypertension into hypertensive heart and/or renal disease; other definite consequences of untreated hypertension, including heart attacks and strokes; and other possible consequences of untreated hypertension, which included some forms of ischemic heart disease and non-traumatic intracranial haemorrhage. We excluded from our main analyses all hospitalizations with primary discharge diagnoses of cardiac conditions coded as I50, I51.4–I51.9, and of renal disease coded as N00, N01, N02, N03, N04, N05, N06, N07, N18, N19, N26, because these codes should not be used to indicate heart and renal diseases among hypertensive patients. However, because use of ICD-10 codes may not be consistent with the stated exclusions, we separately provide calculations of the number and costs of hospitalizations associated with these codes.

**Table 1:** Classification of PhilHealth claims by primary diagnosis ICD-10 codes

**Disease Category**	**Diagnosis Codes**
Essential or secondary HTN	Primary diagnosis = I10 or I15; with or without other diagnoses
Hypertensive heart and/or renal disease	Primary diagnosis = I11; I12; I13; with or without other diagnoses
Other definite consequences of untreated HTN	Primary diagnosis = I20.0; I20.8; I20.9; I21; I22; I23; I25; I34.0; I42.0; I42.1; I42.2; I42.9; I46; I47.2; I48; I61; I63; I64; I65; I66; I67.0; I67.2; I67.4; I67.8; I69.1; I69.3; I69.4; I70; I71; I72; I73.9; I74; N28.0; with or without other diagnoses
Possible consequences of untreated HTN	Primary diagnosis = I24; I49.9; I62.9; I69.2; I77.9; I78.9; I79.8; N27; with or without other diagnoses

HTN = hypertension

Claims were extracted electronically from the central PhilHealth data base after they had been adjudicated and the approved amount had been reimbursed. PhilHealth claims contain patient information (identification number, name, address, age, gender, member/dependent status, membership category); admission and discharge dates; primary and up to two secondary diagnoses (coded with International Classification of Diseases 10^th ^edition (ICD-10) codes) [12]; hospital type (public or private) and location; a case severity indicator (ordinary, intensive, or catastrophic), which is based on ICD-10 codes and the service capability of the accredited provider and used to determine maximum payment amounts [13]; and the reimbursed amounts for different hospital services (room and board, medicines, professional fees, laboratory, and operating room costs). Claims data also contain amounts billed by hospitals for each service. Personal identifiers were removed from the data. The Institutional Review Boards of Harvard Pilgrim Health Care and PhilHealth approved the study.

We analyzed claims data in total and at hospitalization and member level, and describe hospitalization frequency and costs reimbursed by PhilHealth by primary discharge diagnosis category. For the subset of patients who had a hypertension-related hospitalization during the first 18 months of the study, we describe hospitalization frequency, costs, and trajectory of disease development over the rest of the follow-up period and estimated the odds of subsequent HTN-related hospitalizations using logistic regression analyses (SAS^® ^version 9.1).

## Results

### Characteristics of hospitalizations

Between July 2002 and December 2005, PhilHealth reimbursed hospitals for 444,628 admissions for hypertension-related diagnoses incurred by 360,016 patients (Table 2). Hospitalizations for essential or secondary hypertension accounted for the largest proportion (42%) of hospitalizations, followed by hospitalizations for definite consequences of untreated hypertension (37%). Overall, most reimbursed hospitalizations occurred among patients 60–79 years old; among government and private sector employees; and in private hospitals. The majority of hospitalizations (60%) were classified as ordinary, which have the lowest reimbursement limit (Table 2).

**Table 2:** Characteristics (n, %) of hypertension-related hospitalizations reimbursed by PhilHealth

	**All HTN-Related Diagnoses**	**Essential or Secondary HTN**	**Hypertensive Heart and/or Renal Disease**	**Other Definite Consequences of Untreated HTN**	**Other Possible Consequences of Untreated HTN**
Hospitalizations, n	444628	186013	86588	166646	5381
Unique patients, n	360016	166794	67469	144415	5161
^m^Age group*, n	444528	185991	86566	166591	5380
0–39	8.1	11.8	4.9	5.5	11.6
40–59	37.8	45.1	35.7	30.8	32.6
60–79	46.0	37.7	50.1	53.3	44.7
80–100	8.1	5.4	9.3	10.5	11.1
Gender, n	444617	186006	86587	166643	5381
Men	46.7	38.9	46.0	55.8	47.5
Women	53.3	61.1	54.1	44.2	52.4
Member status, n	444617	186013	86588	166646	5381
Member	49.5	46.3	50.5	52.5	50.0
Dependent	50.5	53.7	49.5	47.5	50.0
Membership category, n	444628	186013	86588	166646	5381
Government employee	33.6	35.7	33.2	31.4	32.6
Private firm employee	31.0	30.6	27.7	33.0	32.1
Self-employed	10.8	10.4	13.5	9.8	9.6
Retired/pensioner	13.2	9.7	16.2	15.5	13.9
Sponsored/indigent	11.5	13.6	9.4	10.3	11.8
^m^Hospital type, n	435910	181480	85108	164159	5163
Public	24.8	24.0	22.8	26.9	22.0
Private	75.2	76.0	77.2	73.1	78.0
^m^Hospital location^†^, n	443703	186013	86588	165786	5316
NCR	20.4	12.0	20.5	29.9	17.3
North	18.9	20.7	17.3	17.5	27.1
Central	37.8	40.2	40.3	34.1	31.4
South	22.9	27.2	21.8	18.6	24.2
Length of stay – total (days) – median	1834589 3	549741 2	305705 2	957101 4	22042 3
^m^Medical case type, n	444628	186013	86588	166646	5381
Catastrophic	14.7	1.3	21.1	26.2	14.6
Intensive	25.9	9.1	22.8	45.9	36.6
Ordinary	59.5	89.6	56.1	28.0	48.8
Surgery performed, n	444628	186013	86588	166646	5381
No	93.9	98.5	84.6	93.4	96.3
Yes	6.1	1.5	15.4	6.6	3.7

^m^Some claims were missing data for this variable; *Based on age at admission date; ^†^Location variables refer to major areas of the Philippines; HTN = hypertension

### Costs of hospitalizations

PhilHealth reimbursed US $56 million to hospitals for hypertension-related care during 3.5 years (Table 3), 34% of the total amount claimed by hospitals for this care. The largest part of the total reimbursement (34%) was spent on medicines administered in the hospital. Median cost per hospitalization was US $74 overall, with the lowest median cost ($57) for hospitalizations for essential or secondary hypertension. Hospitalizations for hypertensive heart and renal disease (median cost of $68) accounted for 15% of hypertension-related expenditures, while hospitalizations for other definite sequelae of hypertension (median cost of $130) accounted for the largest share of expenditures (60%).

**Table 3:** Hypertension-related hospitalization costs* reimbursed by PhilHealth

**Cost Categories**	**All HTN-Related Diagnoses**	**Essential or Secondary HTN**	**Hypertensive Heart and/or Renal Disease**	**Other Definite Consequences of Untreated HTN**	**Other Possible Consequences of Untreated HTN**
Total Costs Reimbursed					

Total	55629906	13266422	8504060	33109546	749878
Median	74	57	68	130	84
Interquartile range	(47, 136)	(40, 81)	(44, 112)	(72, 252)	(52, 151)

Room & board					

Total	12144111	3490435	1903868	6598794	151013
Median	17	15	17	29	17
Interquartile range	(11, 31)	(9, 24)	(10, 30)	(16, 51)	(11, 31)

Medicines					

Total	18891088	3743671	2812965	12060518	273935
Median	20	12	19	40	25
Interquartile range	(8, 49)	(6, 26)	(7, 37)	(15, 99)	(10, 58)

Laboratory					

Total	15981484	4045645	2464490	9247897	223452
Median	26	17	20	36	32
Interquartile range	(14, 36)	(9, 32)	(12, 36)	(18, 76)	(15, 42)

Operating room					

Total	741670	86718	151197	494819	8937
Median	0	0	0	0	0
Interquartile range	(0, 0)	(0, 0)	(0, 0)	(0, 0)	(0, 0)

Professional fee					

Total	5205156	1530813	867739	2743100	63505
Median	9	7	9	11	10
Interquartile range	(6, 14)	(5, 11)	(6, 13)	(6, 20)	(5, 15)

* in 2005 US$, using mid-year exchange rates and adjusted for inflation; HTN = hypertension

Since use of ICD-10 codes is not uniform, we also estimated the total number and costs of hospitalizations for cardiac and renal conditions coded with ICD-10 codes that should not be used for hypertensive patients: 14,893 patients incurred 16,890 hospitalizations for cardiac diseases at a cost to PhilHealth of US $2,658,140, while 12,220 patients incurred 114,786 hospitalizations for renal diseases (including chronic renal failure) at a cost of US $7,267,556.

### Repeat hospitalizations

Among 141,103 patients who were discharged with any HTN-related diagnosis during the first 18 months of the study (July 2002 to December 2003), most (80%) had only one hospitalization during the 3.5 year follow-up period, but 20% had at least one other hospitalization. Seven hundred and eight patients (0.5%) incurred 6 or more hospitalizations during the 3.5 years, which constituted more than 5% of hospitalizations among those with sufficient follow-up time for repeat hospitalizations (Table 4). Median hospitalization costs were higher for patients with multiple admissions: $85 for patients with one hospitalization increasing to $1,151 for those with 10 or more hospitalizations (Table 4). Controlling for age, gender, member status and membership category, patients hospitalized for sequelae of hypertension during the first 18 study months had higher odds of re-hospitalization during the study period compared to those hospitalized for treatment of essential or secondary hypertension (hypertensive heart and renal disease OR 1.16, 95% CI 1.12, 1.21; other definite or possible sequelae of hypertension OR 1.12, 95% CI 1.09, 1.15) (Table 5).

**Table 4:** Hospitalization frequency and costs among PhilHealth members with any HTN-related discharges

**Per Member Number of Hospitalizations in 3.5 Years**	**Number (%) of Members n = 141103**	**Number (%) of Hospitalizations n = 189010**	**Total Costs for Hospitalizations and Costs per Member in 3.5 Years (PhP) Total Sum Median Interquartile Range**
1	112107 (79.5)	112107 (59.3)	161222657 85 (54, 157)
2	20637 (14.6)	41274 (21.8)	5906369 188 (119, 341)
3–5	7651 (5.4)	25807 (13.7)	3375467 308 (198, 547)
6–9	479 (0.3)	3301 (1.8)	330315 498 (336, 849)
10+	229 (0.2)	6521 (3.5)	346820 1151 (684, 1979)

Distribution of number of hypertension-related hospitalizations and associated costs (*in 2005 US$, using mid-year exchange rates and adjusted for inflation) in 3.5 years for PhilHealth members with an admission during the first 18 months of the study (July 2002–December 2003)

**Table 5:** Predictors of subsequent hospitalizations among PhilHealth members

**Characteristic**	**Subsequent admissions for HTN-related conditions among those admitted for any HTN-related condition N = 141,384**		**Subsequent admissions for HTN-related conditions among those admitted for essential or secondary HTN N = 60,646**	
	OR	95% CI	OR	95% CI

Illness Category				
Hypertensive heart or renal disease	1.16	1.12, 1.21	n/a	n/a
Definite or possible consequences	1.12	1.09, 1.15	n/a	n/a
Essential/secondary HTN	-	-	n/a	n/a
Age group (years)				
80+	1.80	1.68, 1.94	3.55	3.03, 4.15
60–79	1.47	1.38, 1.56	2.65	2.34, 3.00
40–59	1.86	1.75, 1.97	1.92	1.70, 2.17
<= 40	-	-	-	-
Gender				
Female	1.08	1.05, 1.11	0.84	0.79, 0.89
Male	-	-	-	-
Membership Status				
Dependent	1.05	1.02, 1.09	1.12	1.05, 1.19
Member	-	-	-	-
Membership category				
Government-employee	1.20	1.16, 1.24	1.14	1.07, 1.22
Self-employed	1.56	1.49, 1.64	1.15	1.03, 1.29
Retired	3.78	3.60, 3.96	1.06	0.93, 1.21
Sponsored/indigent	0.95	0.89, 1.01	0.87	0.77, 0.98
Private firm employee	-	-	-	-

Predictors of subsequent hospitalizations during 3.5 years among PhilHealth members with an admission for any and for essential or secondary HTN during the first 18 months of the study (July 2002–December 2003)

Among the 60,659 patients who were admitted during the first 18 months of the study with a diagnosis of essential or secondary hypertension, 9% were hospitalized again within the study period for treatment of sequelae. Median time to first re-hospitalization for sequelae ranged from 178 days to 255 days after the first observed hospitalization for essential or secondary hypertension (Table 6). For these patients, the odds of readmission were higher among older patients compared to younger; dependents compared to members; and government employees and self-employed members compared to privately employed members; and lower for women compared to men; and sponsored/indigent members compared to privately employed members (Table 5).

**Table 6:** Hospitalization trajectory among PhilHealth members with discharges for essential or secondary HTN

**Sequelae**	**Number (%) of Members n = 60659**	**Number (%) of Hospitalizations N = 78109**	**Days to First Hospital Readmission for Sequelae**		
			
			**25^th ^Percentile**	**50^th ^Percentile**	**75^th ^Percentile**
Hypertensive heart disease	1948 (3.2)	2297 (2.9)	85	247	496
Hypertensive renal disease	110 (0.2)	411 (0.5)	60	255	502
Hypertensive heart and renal disease	26 (0.0)	26 (0.0)	68	178	367
Other definite consequences of untreated HTN	3292 (5.4)	3919 (5.0)	59	225	496
Other possible consequences of untreated HTN	154 (0.3)	163 (0.2)	80	211	437

Reasons for subsequent hypertension-related hospitalization during 3.5 years for PhilHealth members with an admission for essential or secondary HTN during the first 18 months of the study (July 2002–December 2003) HTN = hypertension

## Discussion

With almost half a million hospitalizations for hypertension-related conditions costing PhilHealth US $56 million, the burden of inpatient care for treating hypertension and its sequelae and the burden of cost to PhilHealth are substantial. Of the total burden of cost, medicines administered during inpatient stays constituted the largest component of PhilHealth's reimbursement.

A large proportion of hospitalizations (42%, or 185,636 hospitalizations) were for essential hypertension (ICD-10 code I10). These hospitalizations can usually be prevented through appropriate treatment in ambulatory care. Disease progressed within less than one year to the point where hospitalization for sequelae was experienced by 9% of patients with essential and secondary hypertension hospitalized earlier in the study period, for a total of almost 7,000 additional hospitalizations. With appropriate ambulatory care, many of the sequelae of hypertension could be prevented and hospitalizations for such sequelae avoided.

Our study has several limitations, all of which are likely to result in underestimates of the number of hospitalizations and costs of care. The classification of hospital claims relies on ICD-10 diagnosis codes entered at the billing hospital. These codes may not accurately reflect the condition for which a patient was treated during the hospitalization. We conservatively classified hospitalizations based on primary discharge diagnoses to include only hospitalizations with hypertension or sequelae as the primary reason for care. While inclusion of hypertension diagnoses in secondary diagnosis fields may have reflected more correctly the prevalence of hypertension among the study population, it would likely have led to overestimation of costs of inpatient care for hypertension, by including costs for primary reasons of admission other than hypertension. We also focused our main analyses on diagnosis codes specific for essential or secondary hypertension and hypertensive heart and/or renal disease, excluding codes for heart and renal diseases which could be sequelae of hypertension but for which other codes should be used. If even a small percentage of the more than 100,000 hospitalizations with primary diagnosis chronic renal failure (ICD-10 code N18) occurred among patients with hypertension and were attributable to the progression of the disease, the estimates of costs of care reimbursed by PhilHealth for hypertensive patients would be substantially higher.

Because of the limited follow-up time for our study, we underestimate the incidence of sequelae of hypertension over time. Further studies are warranted of the natural progression of hypertension in the Philippines and the downstream costs of failing to control the illness among outpatients.

Our study is based on data from claims submitted to and paid for by PhilHealth. PhilHealth provides first-dollar coverage for up to 45 days of hospitalization per year for members and up to 45 days per year to be shared among dependents [11]. Claims data allowed us to calculate the amount billed by hospitals to PhilHealth and the amount reimbursed by PhilHealth for inpatient care of patients hospitalized for hypertension treatment. The large difference between billed and reimbursed amounts – PhilHealth reimbursed 34% of what was billed – accounts for denied parts of or entire claims but does not consider costs for which claims were never submitted. Therefore, the data do not allow us to estimate the overall cost of care, particularly for sicker patients, who reached the maximum reimbursement for care during a hospitalization or who required more than 45 hospital days during a given year and needed to pay for additional care out-of-pocket. End-stage renal disease patients, for example, require 3 days of hospitalization per week for dialysis; for those, we only captured the first 45 hospitalization days per year. In addition, we are not able to observe costs among patients unable to afford the out-of-pocket share of hospitalization who chose not to present for needed care.

Claims data also do not allow us to assess the impact of hypertension and sequelae on patients and their families. In addition to the negative physical and emotional consequences, patients suffer losses in income due to hospitalizations; incur expenses for paying for inpatient care when maximum reimbursement limits are reached; and may lack affordable access to outpatient medicines that would prevent a worsening of their disease.

Antihypertensive pharmacological therapy has a major impact on health [14]. The higher the blood pressure, the greater is the risk of heart attack, stroke, and kidney disease [5]. Anti-hypertensive medication treatment drastically reduces these risks by between 20% (for heart attacks) and more than 50% (for heart failure) [6]. However, patients can only adhere to pharmaceutical therapy for chronic disease when they can afford it. In 2005, one month of treatment with a beta-blocker (atenolol) purchased in a private pharmacy cost the equivalent of 3 days of wages of the lowest paid government worker (254 pesos in 2005); one month of treatment with an angiotensin-converting enzyme inhibitor (captopril) cost the equivalent of 6 days wages [8]. Many Filipinos do not have the resources of the lowest paid government worker: 14% of the country's 87 million people live on less than $1 per day [15] and 48% live on less than $2 per day [7].

Data from the United States show a 26% reduction in use of antihypertensives when patient copayments for the drugs were doubled and suggest that reducing patients' out-of-pocket costs could increase the use of preventive cardiovascular medications [16]. In fact, recent analyses of costs and benefits of combination pharmacotherapy for secondary prevention after a myocardial infarction among U.S. patients suggest that providing the medications for free (without co-payments) would reduce mortality and reinfarction rates and save $5,974 per patient [17].

In addition to assuring that appropriate diet and exercise are recommended in ambulatory care, an outpatient medicines benefit to facilitate access to affordable antihypertensives could be one policy option to reduce PhilHealth costs for inpatient care for patients with hypertension. One hospitalization for hypertensive heart and/or renal disease costs PhilHealth $68, which could buy thiazide treatment at private retail prices (2005 US $6.19/month) for one patient for 11 months, not considering the lower prices which PhilHealth should be able to negotiate for covered outpatient medicines. An outpatient medicines benefit would also allow PhilHealth to improve quality of care by reimbursing for antihypertensive therapy that follows standard treatment guidelines.

Designing and implementing an outpatient medicines benefit would require careful consideration and understanding of the population of potential beneficiaries; the extent of the benefit; the logistics of implementation; and of its costs and benefits. A pilot study could answer a series of critical questions related to the feasibility, sustainability, and impacts of such a benefit. How could potential beneficiaries of this benefit be identified? Which type, quantity, and frequency of medicines would PhilHealth subsidize, for which members, in part or in whole? How would PhilHealth contract with pharmacies to supply the medications? Could adherence to treatment guidelines be enforced? How could misuse of the benefit be detected and avoided? PhilHealth and its collaborators have begun the design of this type of outpatient medicines benefit pilot study. The goals of this work are to improve access to affordable medicines and to improve the health of an increasing number of Filipinos with chronic conditions covered by PhilHealth.

## Conclusion

Inpatient care for hypertension and its sequelae is costly for PhilHealth. Many hospitalizations should be avoided with continued ambulatory antihypertensive pharmacologic therapy. Our study raises questions as to whether PhilHealth members have access to appropriate outpatient treatment of hypertension, and whether an outpatient medicines benefit may be one policy option for PhilHealth to decrease inpatient admissions and costs.

## Competing interests

Drs. Wagner and Ross-Degnan, Ms. Graves declare that they have no competing interests. Dr. Valera is a Senior Vice President of the Philippine Health Insurance Corporation and receives salary from PhilHealth. At the time of the study, Dr. Shiela Laviña was a paid employee of PhilHealth, under the supervision of Dr. Valera.

## Authors' contributions

AKW, MV, and DRD were responsible for the conception and design of the study. SL was responsible for PhilHealth data extraction. AJG conducted all analyses. AKW drafted the manuscript. All authors read and approved the final manuscript.

## Pre-publication history

The pre-publication history for this paper can be accessed here:


